# Adherence to Guidance About Complementary Feeding and Vitamin D Supplementation in Infants: The Role of Information Sources

**DOI:** 10.1002/nop2.70400

**Published:** 2025-12-21

**Authors:** Suzannah Helps, Gillian Mancz, Taraneh Dean

**Affiliations:** ^1^ School of Dental, Health and Care Professions University of Portsmouth Portsmouth UK; ^2^ School of Health Sciences University of Southampton Southampton UK; ^3^ London South Bank University London UK

## Abstract

**Aim:**

To identify the characteristics of parents with poor adherence to guidelines around introduction of solid foods and supplementation with Vitamin D, and to assess the role of different sources of information in adherence to these guidelines.

**Design:**

A cross‐sectional survey which was part of a birth cohort study following pregnant mothers and their infants in a UK city.

**Methods:**

390 parents who had consented to take part in a birth cohort study were sent a postal questionnaire when their infant was around 6 months old. 220 parents completed this questionnaire about their infants' diet and the sources of information they had used to make decisions about introducing complementary foods to their infant.

**Results:**

Around half (54%) of parents adhered to current guidelines to delay the introduction of solid foods until after their infants were 24 weeks, and adherence to guidelines was positively associated with maternal age and education level as well as with the use of formal information sources such as a Health Visitor or leaflets. Vitamin D supplementation rates of infants by exclusively breastfeeding mothers were low (35%) but increased over time. The internet was a particularly significant source of information used by parents to make feeding decisions for their infants.

**Conclusion:**

In this sample, adherence to guidance around the introduction of solid foods and Vitamin D supplementation was low. Health professionals must develop strategies to communicate these guidelines more effectively.

**Patient or Public Involvement:**

Members of a PPI group were involved in the study design and development of study materials, including marketing materials.

The World Health Organisation (WHO) currently recommends that infants are exclusively breastfed until 6 months of age. At 6 months, solid foods should be introduced to the infant's diet alongside breast milk as breast milk alone is no longer able to meet the nutritional requirements of the infant (World Health Organisation 2001). The WHO guidelines were adopted by the UK in 2003 (Department of Health 2003), further guidelines introduced in 2016 recommend that, in order to prevent rickets (the softening or weakening of bones in children), exclusively breastfed infants should be supplemented with 8.5–10 mcg of Vitamin D daily until 1 year of age. This supplementation is not necessary in formula‐fed infants (who consume 500 mL of formula per day) as infant formula is supplemented with Vitamin D (Scientific Advisory Committee on Nutrition 2016).

However, these recommendations are not always endorsed by professional bodies (e.g., Fewtrell et al. 2017) and many commercially available infant foods are targeted at infants under 6 months (e.g., Gómez‐Martín et al. 2019; Moumin et al. 2020), which can cause uncertainty around the best approach to introduce solid foods to infants for parents and health professionals (Garcia et al. 2019; Neve et al. 2024).

Compliance with the WHO guidelines around the introduction of solid foods remains low in the UK. Only around half of respondents reported following the guidelines and waiting until their infant was 6 months or older to introduce solid foods in The Scottish Maternal and Infant Nutrition Survey in 2017 (46%) and in an online survey of UK parents (55%) (Spyreli et al. 2022). In the Scottish Maternal and Infant Nutrition Survey, younger mothers and mothers living in areas of greater deprivation were more likely to introduce solid foods earlier (Scottish Government 2017).

Very limited research has investigated the extent to which the recommendations about Vitamin D supplementation have been adhered to in the UK. A European‐wide survey reported that the UK had the lowest adherence to vitamin D supplementation in Europe, with adherence rates of 5%–20% (Uday et al. 2017). A small mixed‐methods study including 64 parents of infants aged 4–12 months reported that only 25% of participants gave their infant vitamin supplements and only 31% knew the recommendations around vitamin D (Garcia et al. 2019). Hemmingway et al. (2021) reported that in Ireland only around 30% of parents were fully compliant with Vitamin D supplementation.

Given the limited adherence to recommendations around introduction of solid foods and vitamin D supplementation in the UK, it is important to understand the factors which underlie this relationship. For example, which information sources parents use to determine how they will introduce solid foods to their infants, and how these sources of information affect the decisions they make. Later introduction of solids has been associated with greater use of formal information sources (McAndrew et al. 2012; Moore et al. 2014). Spurlock et al. (2023) identified that mothers prioritised information around infant feeding being accessible and coming from a trusted source.

Identifying the characteristics of mothers with poor adherence to introduction of solid foods or vitamin D supplementation guidelines will clarify where targeted interventions should be most appropriately directed and the role of different sources of information in adherence to these guidelines will identify the most appropriate methods to convey this information.

The aims of the current study are as follows:

1. To determine the characteristics of mothers who do or do not adhere to guidelines around the introduction of solid foods.
2. To determine the effects of accessing information from different sources on the likelihood that parents adhere to guidelines about introduction of solid foods and vitamin D supplementation.

## 1 Methods

### 1.1 Design

The study was a cross‐sectional survey which was sent to participants as part of a birth cohort study.

### 1.2 Recruitment and Eligibility

Participants were part of a longitudinal population‐based birth cohort study. Full ethical approval for the Birth Cohort registry was given by South Central Berkshire B Research Ethics Committee (REC ref.: 15/SC/0008) and all mothers gave informed consent to take part in the study. Pregnant women were recruited into the study during an antenatal screening appointment at the Queen Alexandra Hospital, Portsmouth. The study was designed to gather data on a wide range of factors around the health and development of infants (see Helps et al. 2025); however, this article specifically focuses on the areas of the study related to the introduction of complementary foods in the infants' diets.

### 1.3 Participants

A total of 390 participants consented to take part in the study and waves of data collection occurred antenatally (Wave 0), at birth (Wave 1), when the infant was around 6 months (Wave 2), around 12 months (Wave 3), and around 24 months (Wave 4). Only live births were included in the registry for subsequent follow‐ups. This article reports primarily on data collected from the Wave 2 questionnaire, which relates to the introduction of complementary foods and supplements in the infants' diet. This questionnaire was a self‐completed paper questionnaire, which was sent to the participant with self‐addressed stamped envelopes for return, and reminder questionnaires were sent after 1 month to parents who had not responded. This questionnaire was sent to the infants' parents around the time that the infant reached 6 months of age, and was completed between September 2016 and September 2018.

### 1.4 Measures

#### 1.4.1 Socio‐Demographic and Environmental Characteristics

Parental socio‐demographic data was collected from the mother antenatally, through the Wave 0 questionnaire which was completed with the recruiting research midwife. Data collected included maternal age, marital status, maternal and paternal education level, maternal and paternal employment status, ethnicity and parity of pregnancy.

#### 1.4.2 Introduction of Complementary Foods

A parent reported questionnaire completed in Wave 2 asked parents to report the age in weeks when they first introduced solid foods to their infant; they were asked to report the first three foods that they fed their infant and whether they first fed their infant pureed foods, finger foods or a mix of finger and pureed foods. Parents were also asked whether their infant was fed any dietary supplements and, if so, to specify which supplements. The nutritional information listed in these supplements was analysed to determine whether they included Vitamin D.

#### 1.4.3 Sources of Information

A questionnaire developed by Moore et al. (2012) was used to determine which sources of information parents had used to make decisions about introducing complimentary foods to their infants. Parents were asked to select from a list (including Health visitor, internet, books, mother, grandmother, friends with older children, friends with the same age children, leaflet, GP or other medical professional) which sources of information they had used to make these decisions and to indicate which source had been the most influential in their decision.

### 1.5 Data Analysis

SPSS (IBM, version 26) was used to analyse the data. Categorical variables were expressed as frequency and percentage, and the *χ* ^2^ test was used to test the relationship between categorical variables. *T*‐tests were used to make between‐group comparisons for continuous variables. Logistic regressions were used to determine the impact of using different sources of information on the likelihood that participants adhered to official guidance around delaying the introduction of solid foods until 24 weeks or vitamin D supplementation.

## 2 Results

### 2.1 Participants

220 participants replied to the Wave 2 questionnaire; however, excessive data (> 10%) were missing from 15 questionnaires, so 205 responses were analysed. 104 (51%) infants were male, and the mean age of the infant when the questionnaire was completed was 6.57 months (SD 0.866). Most questionnaires were completed by the mother (*n* = 200), one was completed by the father, and four by both parents together. The majority of respondents were white British (79%), which is representative of the city of Portsmouth.

### 2.2 Introduction of Solid Foods

**FIGURE 1:**
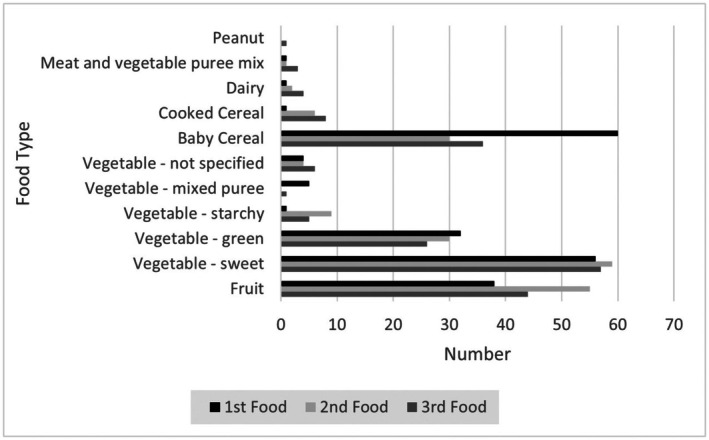
Number of infants introduced to each food type as their 1st, 2nd and 3rd food.

The mean age that infants were introduced to solid foods was 22.6 weeks (SD = 3.6 weeks, range 6–36 weeks). As can be seen from Figure 1, vegetables were most commonly introduced as the infant's first foods, followed by infant cereals and fruits.

### 2.3 Characteristics of Mothers Who Introduce Solid Foods Early or Who Adhered to Recommendations

Table 1 shows that 93 infants were introduced to solids early (before 24 weeks) and 112 were introduced to solids following recommendations. Mothers who introduced solids early were younger and less likely to have attended higher education than mothers who introduced solids following recommendations. There was no difference in paternal education level or in the percentage of parents speaking English as a second language between infants who were introduced to solids early or following recommendations. Parents who followed recommendations were more likely to use finger foods, and were more likely to introduce fruits or vegetables, and less likely to introduce infant cereals as the first food compared to those who introduced solids earlier.

**TABLE 1:** Characteristics of mothers who introduced foods early (before 24 weeks) or adhered to recommendations (24 weeks or later).

	Early	24 weeks or later	
*N* (%)	93 (46%)	112 (54%)	
Maternal age (years)	32.1	33.4	*t*(197) = 2.06, *p* = 0.041
Mothers completed higher education	55 (63%)	83 (75%)	*χ* ^2^(2) = 6.65, *p* = 0.035
Fathers completed higher education	41 (48%)	60 (54%)	*χ* ^2^(2) = 4.03, *p* = 0.134, ns
English as a second language	8 (9%)	6 (5%)	*χ* ^2^(1) = 1.47, *p* = 0.225, ns
Use finger‐food/mixed finger food and purees	39 (42%)	74 (73%)	*χ* ^2^(1) = 18.09, *p* < 0.001
Feeding homemade foods	57 (63%)	73 (73%)	*χ* ^2^(1) = 2.05, *p* = 0.152, ns
Introduced fruit or vegetable as first food	54 (59%)	79 (71%)	*χ* ^2^(1) = 7.470, *p* = 0.006
Introduced infant cereal as first food	36 (39%)	22 (20%)	

### 2.4 Sources of Information Used to Make Decisions About Introducing Solid Foods to Infants

The internet was the most commonly used source of information to make decisions about introducing solid foods to infants and was also most frequently reported to be the most influential source of information used by parents (see Table 2). Health visitors and books were also common sources of information. People who used the internet as a source of information reported finding information about introducing solid foods less consistent (2.94) than those who did not use the internet as a source of information (2.49: *t*(196) = 2.435, *p* = 0.016).

**TABLE 2:** The frequency of different sources of information used to make decisions about introducing solid foods.

	Number (%) reported to use as a source of information	Number (%) reported to be the most influential source of information
Health visitor	109 (55%)	29 (14%)
Internet	146 (75%)	34 (17%)
Books	107 (54%)	30 (15%)
Mother	60 (30%)	11 (5%)
Grandmother	11 (6%)	1 (< 1%)
Friends with same age children	98 (49%)	15 (7%)
Friends with older children	61 (31%)	10 (5%)
Leaflet	62 (31%)	7 (3%)
Medical professionals e.g., GP	25 (13%)	11 (5%)
Other	n/a	27 (13%)
Multiple responses	n/a	26 (13%)

### 2.5 Effects of Accessing Information From Different Sources on the Likelihood That Parents Delay Introduction of Solid Foods to 24 Weeks

A logistic regression was performed to ascertain the effects of accessing information from different sources of information about introduction of solid foods on the likelihood that participants adhered to official guidance and delaying the introduction of solid foods until 24 weeks. The logistic regression model was statistically significant, *χ* ^2^(9) = 31.034, *p* < 0.0001. The model explained 19.7% (Nagelkerke *R* ^2^) of the variance in adherence to guidance and correctly classified 68.6% of cases. People who used a Health Visitor as a source of information were 2.15 times more likely to adhere to guidelines than those who did not; similarly, those who used a leaflet were 2.10 times as likely to adhere to guidelines than those who did not. Those who used their mother as a source of information were less likely to adhere to guidelines than those who did not (72% less likely); similarly, those who used their GP as a source of information were less likely to adhere to guidelines than those who did not (64% less likely).

### 2.6 Effects of Accessing Information From Different Sources on the Likelihood That Parents Adhere to Guidelines About Vitamin D Supplementation

Of the 77 women who were exclusively breastfeeding, 27 (35%) reported that they gave their infants vitamin supplements (12 multivitamins, 15 Vitamin D). There was no difference in age of the women who gave the vitamin supplements compared to those who did not (supplements 33.4 years, no supplements 34.1 years, *t*(73) = 0.663, *p* = 0.509, ns). Mothers who gave their infants supplements were no more likely to have attended Further Education than those who did not (89% vs. 78%, Fishers exact test = 0.355, ns), nor was the father more likely to have attended Further Education (52% vs. 78%, *χ* ^2^ = 0.743, *p* = 0.389, ns).

There was a significant increase in adherence to Vitamin D supplementation from 2016 to 2017 to 2018 (17%: 34%: 64%; Linear by Linear Association *χ* ^2^ = 5.38, *p* = 0.020).

A logistic regression was performed to ascertain the effects of accessing information from different sources of information about the introduction of solid foods on the likelihood that mothers used vitamin D supplements. The logistic regression model was not statistically significant, *χ* ^2^(4) = 0.521, *p* = 0.971 and using any of the sources of information did not increase the likelihood of child vitamin D supplementation.

## 3 Discussion

In this population cohort study, around half of parents (54%) adhered to current guidelines to delay the introduction of solid foods until after their infants were 24 weeks, which is similar to rates described in the Scottish Maternal and Infant Nutrition Survey (2017: 46%). Adherence to these guidelines was positively associated with maternal age and education. Parents who adhered to the guidelines were more likely to use a finger food or mixed finger food and puree approach, and were more likely to introduce fruits or vegetables, and less likely to introduce infant cereals as the first food compared to those who introduced solids earlier.

Also similar to previous research, in the current study, the use of formal information sources such as a Health Visitor or leaflet was associated with higher adherence to these guidelines (e.g., Scottish Government 2017; Moore et al. 2012). Although the participants in the current study do not explicitly state which leaflets they used in making decisions introducing solid foods to their infants, they are likely to be the leaflet given by Health Visitors to mothers of infants—‘Start 4 Life Introducing Solids Foods’ an NHS leaflet which covers the signs of readiness for solids and which first foods to introduce. The information given by the Health Visitors is likely to be evidence‐based and based on NHS recommendations so it is unsurprising that the parents who reported using these sources of information showed greater adherence to the guidelines. Perhaps surprisingly, information from other Health Professionals, such as GPs, was associated with lower adherence to the guidelines. It may be that the other health professionals, who are less likely to be specially trained in working with infants, are less knowledgeable about the guidelines. However, it may be that these health professionals show greater variability in how they interpret the guidelines and give advice about individual management of an infant rather than the general recommendations.

The current study also showed that the internet was particularly significant in parents feeding decisions for their infants. The internet was reported to be the most commonly used source of information to make decisions about infant feeding, and was used by around 75% of respondents. The internet was also reported to be the most influential source of information used by parents to make decisions about feeding their infant. This is similar to other research, which typically reports that around 75% of people use the internet to access health information (Al Muammar et al. 2021). Parents have reported preferring online sources to find information about infant feeding due to their instant nature, and although they report that they hold information given by formal health care providers such as the NHS in high regard, they state that they are less likely to approach health care workers for this information due to accessibility issues (Spurlock et al. 2023).

Young people are more likely to use the internet to seek information about health than older people, but the information on the internet can be inaccurate and of low quality (e.g., Batar et al. 2020). This can cause uncertainty as patients do not know which sources from the internet are reliable, and in the current study, parents who used the internet as a source of information about infant feeding reported finding information to be inconsistent. However, reviews of internet videos typically show that videos shared by health professionals and government organisations are of high reliability, accuracy, and quality (e.g., Inan‐Eroglu and Buyuktuncer 2022). Given the high demand for health information on the internet, health professionals, academics and government organisations need to engage more with internet platforms to provide clear evidence‐based information to patients. This is likely to be particularly effective for sharing health information with younger people.

In the current study, Vitamin D supplementation rates of infants by exclusively breastfeeding mothers was low (35%) but increased over time, which is in line with previous published research (e.g., Scottish Maternal and Infant Nutrition Survey 2017; Uday et al. 2017; Hemmingway et al. 2021). As the number of exclusively breastfeeding mothers in this sample was small (*n* = 77), larger studies will be required to obtain a more accurate figure of the numbers of exclusively breastfeeding mothers who adhere to these guidelines. However, there were no associations between adherence to these guidelines and any parental characteristics, such as age or education, or the use of any source of information, which suggests that these recommendations may be poorly communicated to all mothers. This is supported by the findings of Garcia et al. (2019) where only 31% of mothers knew the recommendations around vitamin D. Further research with health professionals and parents is necessary to determine the barriers that prevent the communication about guidelines around Vitamin D supplementation, and methods that can be employed to improve their communication.

A major strength of the current study was the use of a community cohort sample which eliminated many self‐selecting biases. For example, Moore et al. (2012) and Garcia et al. (2019)'s investigations recruited mothers from parenting websites, and are likely to have over‐representation of mothers who use the internet. The current study recruited mothers at their antenatal appointment and used postal questionnaires for follow up waves of data collection; this removes any self‐selection bias surrounding the internet. The current study also reduced the chances of recall bias as questionnaires were sent to parents around the time they were introducing solid food to their infants rather than relying on recall of past experience. However, limitations of the study are the reliance on parental report which is not always accurate, and particularly in this study parents may over report adherence to guidance (social‐desirability bias). Also, respondents to the study were predominately white and highly educated, which may limit the generalisability of the findings to other groups.

## 4 Conclusions

In this population cohort, adherence to guidelines around infant feeding was poor. Older and more highly educated mothers, and those that used a Health Visitor or leaflet as a source of information, were more likely to delay introduction of solid foods until 24 weeks. As the internet was frequently used and an influential source of information about infant feeding, health professionals and academics should engage more with internet platforms to promote guidelines around infant feeding.

### 4.1 Relevance of Research to Nursing

This research is particularly relevant to health visitors, midwives and other public health nurses who are responsible for promoting public health guidance to families. Given the high demand for health information on the internet, using these methods could provide clear evidence‐based information to large numbers of patients and the public. This is likely to be particularly effective for sharing health information with younger people.

## Author Contributions

All authors were involved in the study design; S.H. and G.M. were involved in data collection. S.H. wrote the first draft of the paper and conducted the analyses; all authors contributed to further drafts.

## Funding

The study was funded by the University of Portsmouth.

## Conflicts of Interest

The authors declare no conflicts of interest.

## Data Availability

The data that support the findings of this study are available on request from the corresponding author. The data are not publicly available due to privacy or ethical restrictions.
